# Special Issue “Deployment of Proteomics Approaches in Biomedical Research”

**DOI:** 10.3390/ijms25031717

**Published:** 2024-01-31

**Authors:** Joaquín Fernández-Irigoyen, Enrique Santamaría

**Affiliations:** Proteomics Platform, Clinical Neuroproteomics Unit, Navarrabiomed, Hospitalario Universitario de Navarra (HUN), Navarra Institute for Health Research (IDISNA), Universidad Pública de Navarra (UPNA), Irunlarrea 3, 31008 Pamplona, Spain

Many angles of personalized medicine, such as diagnostic improvements, systems biology [1], drug screening/mechanisms of action [2,3] and bioinformatics, require the deployment of quantitatively precise human proteomics datasets to make omics analysis actionable at a clinical level [4,5,6]. Biomedical proteomics implements protein research to elucidate disease-affected proteotypes and to determine protein features such as origin, localization [7,8], interactions [9,10,11], posttranslational modifications [12,13,14,15] and protein imbalance [16,17] in human malignancies. Multiple contributions were submitted to this Special Issue, with four original articles and two reviews being accepted for publication. These contributions cover different fields of precision proteomics: (i) autoantibody profiling (two articles), (ii) microbial computational proteomics (one article), (iii) chemotherapy response (one article) and (iv) oncoproteomics (two reviews).

Proteomics has emerged as a powerful approach to characterize the serological/plasmatic autoantibody profiles for the diagnostic identification of accurate predictive biomarkers or therapy responses, with the aim of obtaining clinical benefits [18]. Geroldinger-Simic et al. performed a proteotype-wide analysis in the context of systemic sclerosis (SSc), a rare autoimmune systemic disease that leads to a decreased quality of life and chance of survival, due to fibrotic and inflammatory events, as well as vascular impairment in the skin and/or vital organs. Specifically, they applied a combination of untargeted autoantibody screening on a planar antigen array platform (which included 18,000 proteins represented by 42,000 antigens) and a targeted antigen bead array to detect autoantibodies associated with fibrosis in the plasma from SSc patients. From the eleven differential autoantibodies detected in SSc patients relative to the control population, eight of them corresponded to fibrosis-associated proteins, and anti-phosphatidylinositol-5-phosphate 4-kinase type 2 beta (PIP4K2B) and anti-AKT serine/threonine kinase 3 (AKT3) antibodies were proposed to be molecules associated with skin and lung fibrosis in SSc patients, with potential as candidates to be further validated in the SSc diagnostic field [19]. Following a similar approach, Mescia et al. used in-house developed antigen array platforms to identify antibodies associated with anti-neutrophil cytoplasmic antibody (ANCA)-associated vasculitis (AAV), another rare autoimmune disease that causes inflammatory damage in small blood vessels. In this case, 1561 unique proteins covered by 1743 fragments were monitored in serum samples derived from AAV patients at presentation, remission, and relapse, as well as from healthy controls and from patients with different vasculitis and inflammatory-associated conditions, with the aim of evaluating the specificity of the top candidates identified in AAV. After the untargeted/targeted profiling and data analysis, they discovered a high prevalence of anti-kinesin autoantibodies, showing associations with organ involvement but without variations in terms of disease activity [20].

Another relevant aspect of proteomics in biomedicine is the capacity to obtain direct molecular information associated with major oncological treatments like surgery, radiotherapy or chemotherapy. Colorectal carcinoma is the third common cancer diagnosed worldwide and only 30–50% of patients show positive results under combinatorial therapies; chemoresistance is, therefore, a major clinical problem in the treatment of this metastatic cancer. Tam et al. performed mass spectrometry-based proteomics workflows (label-free based) to characterize protein metabolic changes associated with chemotherapy responses between folinic acid and oxaliplatin (FOLFOX)-resistant and wild-type colorectal cancer cells. Analyzing the differential proteomic datasets, the authors pointed out that the up-regulation of the ribosomal process and actin cytoskeleton could be valuable pathways to exploit in order to obtain successful improvements in the treatment efficacy of chemoresistant colorectal tumors [21]. In addition, this Special Issue includes a pair of reviews about the potential applications of proteomics in different aspects of oncology. Zhang et al. outlined the relevance of alternative lengthening of telomeres (ALT) as a mechanism to preserve telomeres, based on the use of the homologous recombination-based pathway by cancer cells. Specifically, they overviewed ALT regulation by shelterin components (like TRF1 and TRF2), which are involved in telomere localization and replication [22]. Felipez et al. focused on the potential exploration of gastric juice as a liquid biopsy enriched in disease-specific biomarkers, in the context of gastric cancer. In this review, the authors compiled all type of potential biomarkers proposed in gastric juice, considering not only proteomic-based biomarkers but also genetic biomarkers, based on non-coding RNAs, microRNAs, piRNAs, lncRNAs and DNA. They highlighted the potential application of multiplexed antibodies (proximity extension assay (PEA) technology from Olink^®^, Uppsala, Sweden) and multiplexed nucleic acid aptamers (SomaScan^®^ aptamer-based technology from SomaLogic; Boulder, CO, USA) [23,24,25,26,27,28,29] as a complementary approach to mass spectrometry, to establish robust clinical proteomic pipelines [30].

During recent years, an additional expansion of biomedical proteomics has been the study of protein inventories derived from microbial communities, as well as their functional activity and dynamic composition [31,32,33]. In this field, the continuous evaluation and testing of bioinformatic pipelines for mining the acquired datasets is pivotal to obtain confident data, as well as to potentially improve LC-MS/MS strategies. Mappa et al. proposed multiple tandem mass spectrometry datasets, recorded on an artificial reference consortium comprising 24 bacterial species, covering 20 different genera and 5 bacterial phyla. This dataset (“Mix24X”) allows access to specific bacterial proteomes, helping to evaluate the assignment strategy of LC-MS/MS spectra derived from highly complex mixtures. In addition, the authors recommended the use of Mix24X to monitor proteotyping pipelines associated with the elucidation of densely genome-sequenced genera and species or closely related microorganisms [34].

To sum up, all these works point out the capacity and opportunities of different proteomic pipelines to generate innovative biomedical knowledge at fluid, tissular and microbial levels, which is necessary to filling the gaps toward the implementation of precision proteomics in the biomedical area.
